# Oral pretreatment with *Escherichia coli* Nissle 1917 enhances the host's defense against influenza A virus infection

**DOI:** 10.1002/mlf2.70050

**Published:** 2025-12-27

**Authors:** Di Wang, Longhai Yu, Qi Lu, Meiqing Han, Baikui Wang, Xianqi Peng, Min Yue, Yan Li

**Affiliations:** ^1^ MOA Key Laboratory of Animal Virology & Zhejiang Provincial Engineering Research Center of Animal Biological Products Zhejiang University College of Animal Sciences Hangzhou China; ^2^ Hainan Institute of Zhejiang University Sanya China; ^3^ State Key Laboratory for Diagnosis and Treatment of Infectious Diseases, National Clinical Research Center for Infectious Diseases, National Medical Center for Infectious Diseases, The First Affiliated Hospital Zhejiang University School of Medicine Hangzhou China

**Keywords:** *Escherichia coli* Nissle 1917, gut microbiome, influenza A virus, metabolite, pipecolic acid

## Abstract

Influenza A viruses (IAVs) pose a significant threat to global health, causing annual epidemics and occasional pandemics with substantial morbidity and mortality. Despite the availability of vaccines and antiviral therapies, the development of novel preventive and therapeutic strategies remains a critical research focus. In this study, we evaluated the protective effects of orally administering *Escherichia coli* Nissle 1917 in IAV‐infected mice and elucidated its mechanisms of action by analyzing cecal microbiota and plasm metabolome profiles. Oral administration of *E. coli* Nissle 1917 alleviated respiratory symptoms, reduced weight loss, and mitigated pathological injury in mice infected with H9N2 or H1N1 IAV. These protective effects were mediated through the modulation of gut microbiota diversity, which increased the abundance of *Bacteroides* and *Akkermansia*, correlating with elevated pipecolic acid levels and ultimately aiding in defense against IAV infection in mice. Notably, we identified that the circulating metabolic molecule pipecolic acid plays a significant role in fighting IAV infection. Our findings suggest the potential usefulness of *E. coli* Nissle 1917 or pipecolic acid in influenza prevention.

## Impact statement

In our pursuit of novel or universal antiviral strategies against influenza, we found vigorous anti‐influenza activity of orally administered *Escherichia coli* Nissle 1917 in a mouse model. The protective effects are associated with alterations in the host's gut microbiota and metabolism, as well as the modulation of cytokine expression induced by *E. coli* Nissle 1917 administration. Elevated levels of specific bacteria, including *Bacteroides* and *Akkermansia*, may enhance the secretion of pipecolic acid, thereby contributing to the defense against influenza virus infection. Importantly, our study underscores the potential of *E. coli* Nissle 1917 and pipecolic acid as promising agents for influenza prevention.

## INTRODUCTION

A century ago, the 1918 influenza pandemic outbreak emerged as one of the most severe epidemics in modern history. Within approximately 18 months after the end of World War I, around 50 million people died from this viral infection^1^, ^2^. The repercussions of this pandemic have reverberated throughout the past century, consistently affecting the health of animals and humans globally in diverse ways. Its unpredictable ability to cross species barriers has left a lasting impact, with a high fatality rate remaining a grim reality^3^, ^4^.

Influenza A virus (IAV) is a segmented negative‐strand RNA virus. The IAV genome frequently mutates during the replication cycle due to the absence of proofreading mechanisms associated with the virally encoded RNA‐dependent RNA polymerase^5^. Also, the genomes of different viruses are prone to recombination. The mutation and recombination often result in antigen shift and antigen drift. This characteristic enables it to “jump” between different hosts, increases its ability to bind to human receptors, and expands the host spectrum for viruses^6^. This characteristic also enables the influenza virus to resist drugs developed against it. The first‐generation influenza antiviral drugs now have minimal effectiveness, while newer classes of drugs, including the neuraminidase inhibitors oseltamivir (Tamiflu^®^) and zanamivir (Relenza^®^), face challenges in terms of drug resistance^7^. The pandemic potential of influenza viruses continues to increase, as do concerns about widespread transmission. For instance, the infection of highly pathogenic avian IAV H5N1 in dairy cows in North America in March 2024, along with the efficient cow‐to‐cow transmissions, raises the potential of cow‐to‐human transmission^8^, ^9^, ^10^. The greatest hope lies in preventing influenza. Pursuing novel preventive and therapeutic strategies remains a critical area of research.

The gut microbiota, a complex and dynamic ecosystem comprising trillions of microorganisms, has been increasingly recognized for its far‐reaching effects on host health, including immune system development, metabolic function, and even behavior. The concept that the gut microbiota could influence susceptibility to and outcomes of viral infections is supported by evidence from both human and animal studies. For instance, alterations in gut microbiota composition have been associated with increased susceptibility to respiratory infections and more severe disease outcomes^11^, ^12^. Furthermore, modulation of the gut microbiota through interventions such as probiotics has shown promise in enhancing host resistance to viral infections^13^, ^14^.

*Escherichia coli* Nissle 1917 (EcN), a Gram‐negative bacterium in probiotics, was isolated in 1917 from the stool of a German soldier who was infected with *Shigella* but showed no symptoms of enterocolitis^15^. This strain is non‐pathogenic and lacks common virulence factors. EcN has been shown to possess some unique prebiotic attributes, including bacteriocins, adhesins, and iron acquisition systems^16^. Substantial evidence has indicated that EcN can improve health conditions, such as treating intestinal disorders (e.g., diarrhea, ulcerative colitis, and inflammatory bowel disease [IBD])^15^, ^17^, ^18^, ^19^, ^20^, strengthening the intestinal barrier against pathogens^21^, ^22^, ^23^, and enhancing innate immune functions in the host^24^. Given its well‐documented ability to modulate both local and systemic immune responses, EcN is an ideal candidate for exploring its potential protective effects against IAV infection. Supporting this, Huang et al. recently demonstrated that intranasal administration of probiotic EcN activates innate immunity in the respiratory tract and provides immediate protection against influenza virus infection^25^. However, it remains unclear whether oral administration of EcN elicits similar effects. This study aims to investigate the protective efficacy of orally administered EcN and its metabolite molecule against influenza virus infection using a murine model.

In this study, we investigated whether EcN can protect mice against IAV infection. The results showed that oral administration of EcN exerted its protective effects by modulating the composition of the intestinal microbiota and host metabolisms. In addition, the metabolic analysis revealed that among the significantly altered metabolites, pipecolic acid was identified and verified as a potential candidate for preventing influenza.

## RESULTS

### Oral pretreatment of EcN confers protection against IAV in mice

**Figure 1:**
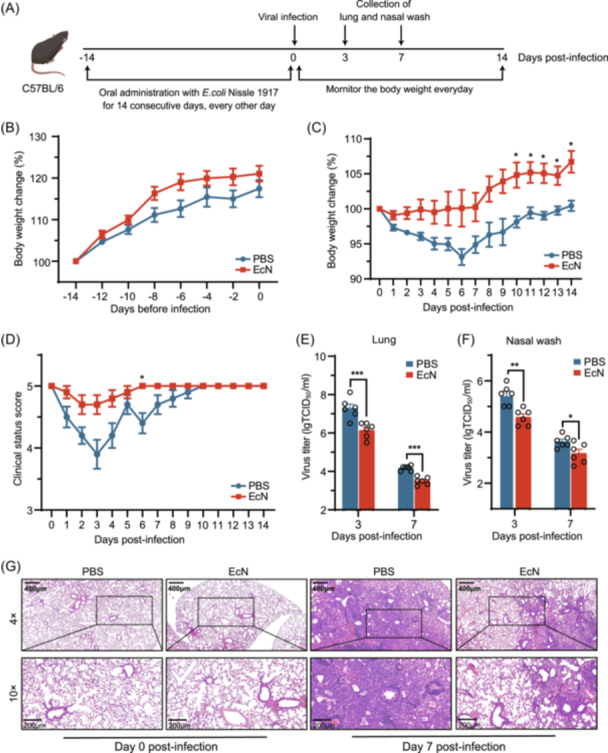
Oral pretreatment of *Escherichia coli* Nissle 1917 (EcN) confers protection against IAV H9N2 Ch01 infection in C57BL/6 mice. (A) Schematic illustration of the experimental design. Female C57BL/6 mice were orally administered phosphate‐buffered saline (PBS) or EcN every other day for 14 consecutive days before intranasal infection with IAV H9N2 Ch01 (10^4.5^ TCID_50_). (B) Daily body weights of mice measured before IAV H9N2 Ch01 infection and after the oral administration of PBS or EcN. Data were normalized to the initial weight of each mouse. A total of 20 mice per group were included in these measurements. (C) Daily body weights of mice measured after IAV H9N2 Ch01 infection. Data were normalized to the initial weight of each mouse (*n* = 10 mice per group). (D) Clinical symptoms recorded and scored after IAV H9N2 Ch01 infection. The criteria were as follows: 5. health (no clinical symptoms); 4. mild (slightly ruffled fur); 3. moderate (ruffled fur and hunching); 2. severe (ruffled fur, hunching, and shivering); and 1. moribund (no reaction to the stimulation) (*n* = 10 mice per group). (E, F) Virus titers in the lungs (E) and nasal wash (F) determined at the indicated time points post‐infection using the TCID_50_ assay (*n* = 6 mice per group). (G) Histological features in the PBS and EcN groups displayed with hematoxylin and eosin (H&E) staining at 0 and 7 days post‐infection (dpi). Representative images are shown at 4× and 10× magnification. All experiments were performed at least three times under similar conditions and yielded consistent results. Data are presented as means ± standard error of mean (SEM). *p < 0.05; **p < 0.01; and ***p < 0.001 (two‐tailed Student's *t*‐test).

To investigate the protective effects of oral administration of EcN against IAV‐related clinical symptoms, 20 C57BL/6 mice per group were treated with EcN or PBS for 14 days before viral challenge with influenza virus A/Mink/China/01/2014(H9N2) (referred to as H9N2 Ch01). Clinical symptoms were monitored, and body weights were recorded (Figure 1A). Baseline body weights remained unchanged during the 14‐day pretreatment period, indicating no growth‐promoting effects of EcN (Figure 1B). Following IAV H9N2 Ch01 infection, PBS‐treated mice experienced 6.87% ± 1.14% loss in body weights, compared to only 0.48% ± 1.7% loss in body weights in EcN‐treated mice (Figure 1C). All PBS‐treated mice showed pronounced clinical signs of infection, including weight loss, anorexia, shivering, and rough hair. In contrast, EcN‐treated mice showed significantly milder symptoms (Figure 1D). Concomitantly, virus titers in both lung and nasal washes of EcN‐treated mice were significantly lower than those of PBS‐treated mice at 3 and 7 dpi (*p* < 0.01) (Figure 1E,F). Histological examination showed that PBS‐treated mice had inflammatory cell infiltration, necrosis, increased foci, congestion, alveolar collapse, and disrupted lung tissue architecture at 7 dpi, which was markedly more severe than EcN‐treated mice (Figure 1G). These findings suggest that pretreatment of EcN may confer protection against IAV H9N2 infection in mice.

**Figure 2:**
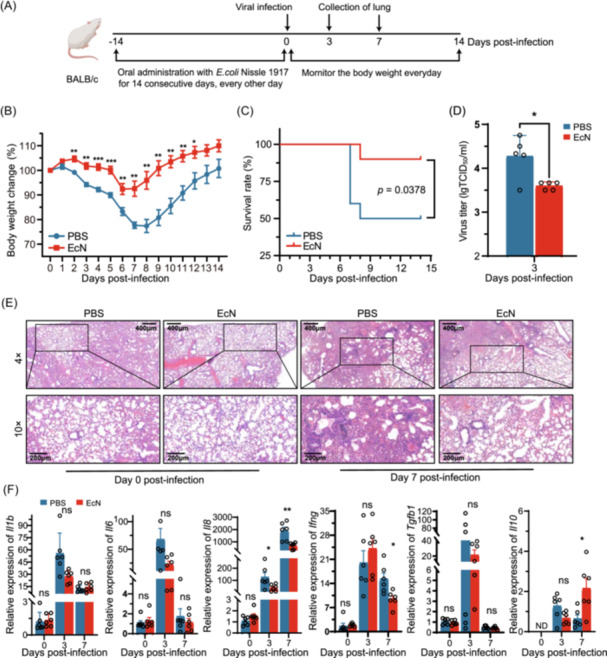
Oral pretreatment of EcN confers protection against IAV H1N1 Ca04 infection in BALB/c mice. (A) Schematic illustration of the experimental design. Female BALB/c mice were orally administered PBS or EcN every other day for 14 consecutive days before intranasal infection with IAV H1N1 Ca04 (10^4.5^ TCID_50_). A total of 19 mice per group were included in these experiments. (B) Body weights of mice measured daily post IAV H1N1 Ca04 infection for 14 days. Data were normalized to each mouse's initial weight. A total of 10 mice per group were included in these measurements. (C) The survival curves were compared using the Gehan–Breslow–Wilcoxon test. (D) Virus titers in the lungs determined on Day 3 post IAV H1N1 Ca04 infection using the TCID_50_ assay (*n* = 5 mice per group). (E) Histological sections of the lungs at the indicated time points post‐infection displayed with H&E staining. Representative images are shown at 4× and 10× magnification. All experiments were performed independently at least three times under similar conditions. (F) Relative expression of IL‐1β, IL‐6, IL‐8, IFN ‐γ, TGF‐ β, and IL‐10 in the lungs collected at 0, 3, and 7 dpi measured by RT‐qPCR (*n* = 6 mice per group). All experiments were performed at least three times under similar conditions and yielded consistent results. Data are presented as means ± SEM. *p < 0.05; **p < 0.01; and ***p < 0.001 (two‐tailed Student's *t*‐test). ND, under‐detectable.

To assess strain‐independent protection, we infected BALB/c mice with influenza virus A/California/04/2009(H1N1)^26^ (referred to as H1N1 Ca04) after EcN administration and monitored body weights and survival (Figure 2A). No significant difference was observed in the body weight changes of the BALB/c mice after EcN administration (Figure S1). There was a reduction in body weight loss after the H1N1 Ca04 virus infection, but the loss was much milder in EcN‐treated mice than in the PBS‐treated mice (Figure 2B). The mortality rate in the EcN‐treated group was 10%, significantly lower than the 50% mortality rate in the PBS‐treated group (*p* = 0.0378) (Figure 2C). Viral titration results showed that EcN administration significantly reduced the virus titers in the lungs at 3 dpi to approximately one‐fifth of those in the PBS‐treated group (*p* = 0.0192) (Figure 2D). Histological analysis at 7 dpi showed that PBS‐treated mice had lung vascular dilation, congestion, extensive inflammatory cell infiltration, large inflammatory foci, disrupted alveolar structure, and minor exudate accumulation, whereas EcN‐treated mice maintained relatively intact alveolar structure with limited inflammatory cell aggregations (Figure 2E). These results demonstrate that EcN confers protective effects against both H1N1 and H9N2 influenza subtypes in murine models.

### Oral pretreatment of EcN modulates cytokine expression during IAV infection in vivo

Given the critical role of inflammatory cytokines in the host immune response to IAV infections^27^, we investigated whether EcN pretreatment modulates pulmonary cytokine and chemokine expression following viral infections. Expression levels of IL‐6, IL‐1β, IL‐8, IFN‐γ, TGF‐β, and IL‐10 in lung tissue were quantified at 0, 3, and 7 dpi of H1N1 Ca04. IL‐8, a cytokine that attracts and activates neutrophils, were significantly lower in the EcN‐treated mice compared to the PBS‐treated mice at 3 and 7 dpi. Additionally, IFN‐γ, a cytokine associated with the inflammatory response and tissue damage, was significantly reduced in the EcN‐treated mice at 7 dpi. Conversely, the anti‐inflammatory cytokine IL‐10 showed markedly higher levels in the EcN‐treated mice at 7 dpi (Figure 2F). These findings suggested that EcN pretreatment modulates the inflammatory response following IAV H9N2 Ch01 infection, potentially mitigating excessive inflammatory cell infiltration and tissue damage while promoting the resolution of inflammation. This immunomodulatory effect may contribute to improved disease outcomes after IAV infection.

### Oral pretreatment of EcN modulates the gut microbiota composition in mice

**Figure 3:**
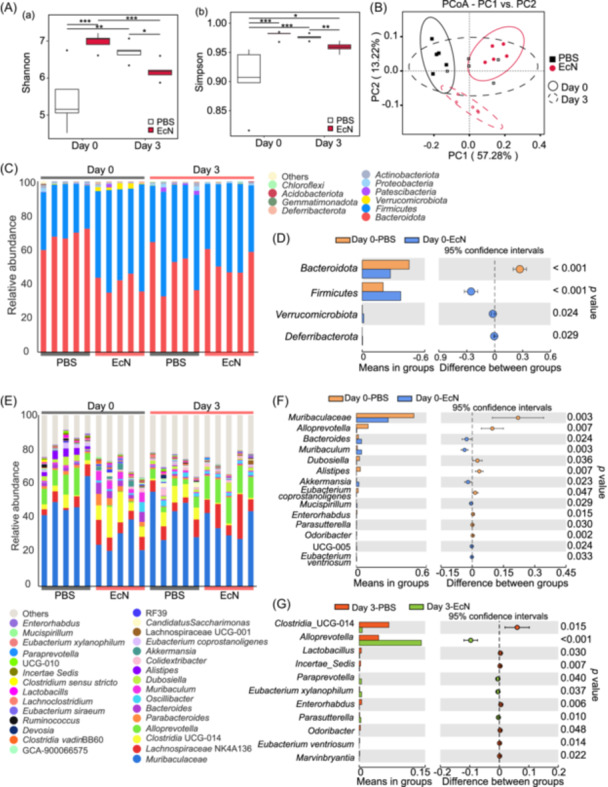
Oral pretreatment of EcN modulates gut microbiota composition. Cecum samples were collected at 0 and 3 dpi and subjected to 16S rDNA sequencing. (A) α‐diversity assessed using the Shannon (a) and Simpson (b) indices (*n* = 5). *p < 0.05; **p < 0.01; ***p < 0.001. (B) β‐diversity assessed using principal coordinate analysis (PCoA) analysis based on weighted UniFrac distance. Each plot represents one sample (*n* = 5). (C) Relative abundance of the gut microbiota at the phylum level in the fecal sample from IAV‐infected mice at 0 and 3 dpi. (D) Differential abundance of bacteria at the phylum level between Day 0‐PBS and Day 0‐EcN groups analyzed using a Student's *t*‐test. (E) Abundance of gut microbiota at the genus level in the fecal sample from IAV‐infected mice at 0 and 3 dpi. (F) Differential abundance of bacteria at the genus level between Day 0‐PBS and Day 0‐EcN groups. (G) Differential abundance of bacteria at the genus level between Day 3‐PBS and Day 3‐EcN groups.

Given the established role of gut microbiota in antiviral immunity^28^, ^29^, ^30^, we characterized the intestinal microbiota profile by 16S rDNA sequencing of cecal contents from PBS‐ and EcN‐treated C57BL/6 mice before and after IAV H9N2 Ch01 infection. A total of 3860 amplicon sequence variants (ASVs) were identified (Figure S2A). Alpha diversity was assessed using four metrics: Shannon index (richness and evenness) (Figure 3A‐a), Simpson index (evenness) (Figure 3A‐b), species accumulation boxplot (richness) (Figure S2B), and rank abundance (richness and evenness) (Figure S2C). Statistically significant increases in Shannon and Simpson indices were observed in EcN‐treated mice compared to the PBS‐treated mice on Day 0 (*p* < 0.001), indicating that EcN treatment enhances gut microbiota diversity and richness. However, by Day 3 post IAV infection, EcN‐treated mice showed reduced microbiota diversity compared to PBS‐treated mice, as evidenced by two indices (*p* < 0.05 or *p* < 0.01) (Figure 3A). Beta diversity, which evaluates the similarity of community structure between groups^31^, was assessed using principal coordinate analysis (PCoA) based on the weighted UniFrac distance. PCoA showed clear separations among treatments (EcN or PBS) and time points (0 or 3 dpi) (Figure 3B), demonstrating that EcN pretreatment significantly altered gut microbiota composition, whereas IAV infection substantially modulated gut microbiota composition.

**Figure 4:**
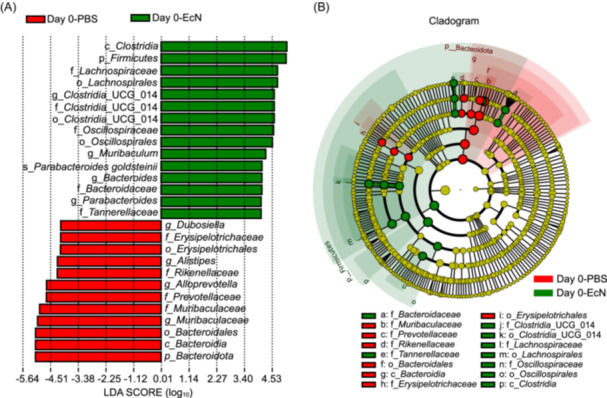
Taxonomic differences between the fecal microbiota of EcN‐ and PBS‐treated mice. (A) Differentially abundant taxa between the PBS and EcN groups at 0 and 3 dpi identified using linear discriminant analysis effect size (LEfSe). Only taxa surpassing the significant LDA threshold value of > 3.5 are presented. Statistical analyses were performed using LEfSe software (Version 1.0). (B) Abundance patterns of bacterial taxa in each data set assessed using the circular cladogram generated by LEfSe.

We analyzed the relative abundance of the top 10 bacterial phyla, including *Bacteroidota*, *Firmicutes*, *Verrucomicrobiota*, *Proteobacteria*, *Patescibacteria*, *Actinobacteriota*, *Deferribacterota*, *Gemmatimonadota*, *Acidobacteriota*, and other taxa (Figure 3C). Before IAV infection, the PBS‐treated mice showed relative abundances of *Bacteroidota*, *Firmicutes*, *Actinobacteriota*, and *Verrucomicrobiota* at 67.42%, 30.06%, 0.88%, 0.74%, respectively. EcN pretreatment altered the bacterial composition, with the relative abundances of *Firmicutes*, *Bacteroidota*, *Verrucomicrobiota*, and *Deferribacterota* at 55.45%, 40.46%, 2.22%, and 0.53%, respectively. The *Firmicutes*/*Bacteroidota* (F/B) ratio, a key indicator of microbial changes^32^, ^33^, ^34^, was significantly higher in EcN‐treated mice compared to PBS‐treated mice on Day 0 post‐infection (*p* = 0.0002). EcN treatment also increased the relative abundance of *Verrucomicrobiota* and *Deferribacterota* compared to the PBS (*p* < 0.05) (Figure 3D). At the genus level, EcN significantly increased relative abundances of *Bacteroides*, *Muribaculum*, *Akkermansia,* and *Mucispirillum* (*p* < 0.05), while decreasing the relative abundances of *Muribaculaceae*, *Alloprevotella*, *Dubosiella*, and *Alistipes* (*p* < 0.05) (Figure 3E,F). Genus‐level analysis further demonstrated the dynamic effects of EcN treatment over time. The EcN‐treated mice showed a significant increase in the relative abundance of genera such as *Alloprevotella*, *Paraprevotella*, and *Eubacterium xylanophilum*, whereas the PBS‐treated mice showed higher relative abundances of genera like *Clostridia*_UCG‐014 and *Lactobacillus* (Figure 3G). These compositional shifts may be attributed to the absence of EcN supplementation in the PBS group. Linear discriminant analysis effect size (LEfSe) analysis identified significant differences in ASVs between the EcN‐ and PBS‐treated mice at 0 dpi (*p* < 0.05) (Figure 4). *Firmicutes* phylum, *Clostridia* class, and *Bacteroides* genus were significantly enriched in the EcN group, while *Dubosiella*, *Muribaculaceae*, and *Alistipes* genera were more abundant in the PBS‐treated mice.

### EcN modulates plasma metabolites in mice

**Figure 5:**
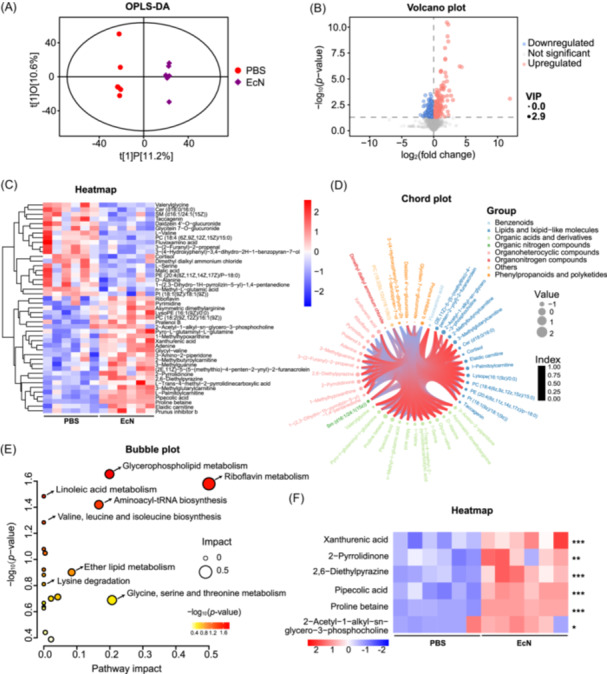
EcN modulates plasma metabolites. (A) The OPLS‐DA score plot showing a significant global metabolic distinction in the plasma of PBS‐treated mice compared to EcN‐treated mice at 0 dpi. Each plot represents a single sample (*n* = 6). (B) Volcano plot in positive mode illustrating differential metabolites between EcN‐ and PBS‐treated mice at 0 dpi. (C) The heatmap showing 45 differential metabolites in EcN‐treated mice compared to PBS‐treated ones at 0 dpi. (D) The metabolites categorized as follows: (1) benzenoids, (2) lipids and lipids‐like molecules, (3) organic acids and derivatives, (4) organic nitrogen compounds, (5) organoheterocyclic compounds, (6) organonitrogen compounds, (7) unclassified, and (8) phenylpropanoids and polyketides. (E) Pathways inferred based on metabolites in positive mode for EcN versus PBS. (F) The heatmap depicting the differentially upregulated metabolites in the plasma of mice treated with EcN compared to PBS at 0 dpi. *p < 0.05; **p < 0.01; ***p < 0.001.

The gut–lung axis is a communication network in which metabolites (e.g., short‐chain fatty acids) modulate immune responses and inflammation to influence respiratory health^35^. To investigate the metabolic mechanisms underlying EcN‐mediated antiviral protection, we used liquid chromatography with tandem mass spectrometry (LC‐MS/MS)‐based untargeted metabolomics on plasma samples from C57BL/6 mice pretreated with EcN or PBS for 14 days (six mice per group). The plasma metabolome reflects the host's physiological state and its interaction with the gut microbiota. Compared to PBS‐treated mice, EcN treatment significantly altered the metabolic profile, as demonstrated by robust orthogonal projections to latent structures‐discriminant analysis (OPLS‐DA) (*R* ^2^ *Y* = 0.998, *Q* ^2^ = 0.307) (Figure 5A), indicating a substantial impact of EcN treatment on the overall metabolism. A total of 443 metabolites in positive ionization mode were identified and quantified, with 206 metabolites upregulated and 237 metabolites downregulated (Figure 5B, Table S1). Hierarchical cluster analysis revealed 44 significantly altered metabolites (Figure 5C). These included one benzenoid, thirteen lipids and lipids‐like molecules, thirteen organic acids and derivatives, one organic nitrogen compound, eleven organoheterocyclic compounds, one organonitrogen compound, four phenylpropanoids and polyketides, and one unclassified metabolite (Figure 5D). EcN treatment also induced changes in metabolic pathways, including ether lipid metabolism, lysine degradation, riboflavin metabolism, aminoacyl‐tRNA biosynthesis, valine, leucine and isoleucine biosynthesis, glycine, serine and threonine metabolism, glycerophospholipid metabolism, and linoleic acid metabolism in mice (Figure 5E). Using the criteria “fold change > 2 and *p*‐value < 0.05,” six metabolites, including proline betaine, 2‐acetyl‐1‐alkyl‐sn‐glycero‐3‐phosphocholine, 2,6‐diethylpyrazine, xanthurenic acid, pipecolic acid, and 2‐pyrrolidinone, were identified to be significantly enriched in the EcN‐treated mice (Figure 5F). The results suggest that EcN treatment protects mice against IAV by enhancing the production of specific metabolites.

### Correlation between metabolites and microbiota composition following EcN treatment

**Figure 6:**
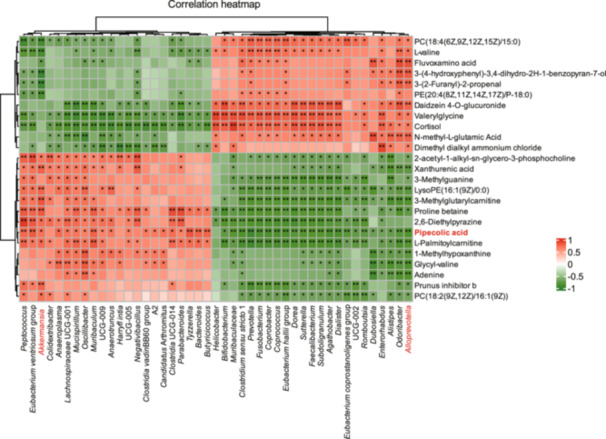
Correlation analysis between metabolites and microbiota composition in EcN‐ treated mice. Heatmap illustrates the correlation between host's plasma metabolites (highlighted in red, including pipecolic acid) and cecal microbiota composition in C57BL/6 mice after 14‐day EcN gavage. *p < 0.05; **p < 0.01.

To elucidate the intricate relationship between gut microbiota and metabolites, we performed a Spearman correlation analysis on the cecal microbiota genera and plasma metabolites of EcN‐treated mice. As shown in Figure 6, increased levels of *Akkermansia* were positively correlated with ten altered metabolites (*p* < 0.05). Conversely, reduced levels of *Alloprevotella* were negatively correlated with thirteen altered metabolites. Among the specific metabolites, pipecolic acid shows a significant positive correlation with *Akkermansia* (*p* < 0.01). Additionally, metabolites such as xanthurenic acid, proline betaine, and 2‐acetyl‐1‐alkyl‐sn‐glycero‐3‐phosphocholine showed increased concentrations following EcN treatment, in contrast to the trends observed for *Muribaculaceae, Alloprevotella,* and *Alistipes*. The results suggest that gut microbiota play a critical role in modulating host metabolism.

### Pipecolic acid confers protection against IAV infection in mice

**Figure 7:**
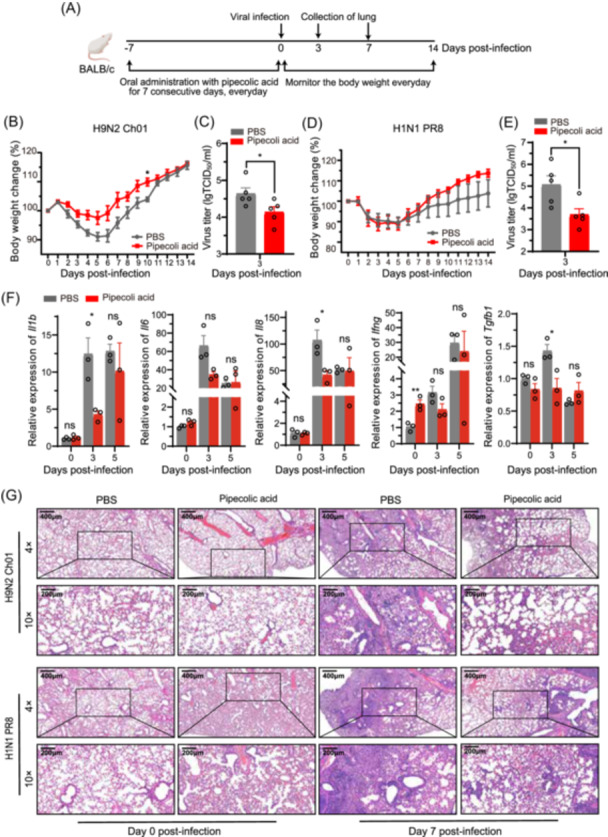
Oral pretreatment of pipecolic acid protects mice against IAV infection. (A) Schematic illustration of the experimental design. Female BALB/c mice orally administered PBS or pipecolic acid once daily for 7 consecutive days before intranasal infection with IAV H9N2 Ch01 (10^4.5^ TCID_50_) or H1N1 PR8 (10^4.5^ TCID_50_). (B) Body weights measured daily after IAV H9N2 Ch01 infection for 14 days (*n* = 6 mice per group). (C) Virus titers in the lungs determined on Day 3 post IAV H9N2 Ch01 infection using the TCID_50_ assay (*n* = 5 mice per group). (D) Body weights recorded daily post IAV H1N1 PR8 infection (*n* = 6 mice per group). (E) Virus titers in the lungs assessed on Day 3 post IAV H1N1 PR8 infection using the TCID_50_ assay (*n* = 5 mice per group). (F) Relative expression levels of *IL‐1β*, *IL‐6*, *IL‐8*, *IFN ‐γ*, *TGF‐ β*, and *IL‐10* in the lungs collected at 0, 3, and 5 dpi measured by RT‐qPCR (*n* = 5 mice per group). (G) Histological sections of the lungs at the indicated time points post‐infection stained with H&E. Representative images are displayed at 4× and 10× magnification. Experiments were performed three times under similar conditions and yielded similar results. Data are presented as means ± SEM. *p < 0.05 (two‐tailed Student's *t*‐test).

To examine whether EcN‐elevated metabolites enhance resistance against IAV, we orally administered pipecolic acid to mice at a dose of 12.5, 25, and 75 mg/kg body weight for 7 days before intranasal challenge with 10^4.5^ TCID_50_ of IAV H9N2 Ch01 (Figure 7A). Lung virus titers at 3 dpi revealed significantly reduction at the dose of 75 mg/kg (Figure S3A), establishing this as the effective dose for subsequent experiments. Before IAV infection, pipecolic acid‐treated mice showed more weight gain than PBS‐treated mice, suggesting no adverse effects of pipecolic acid on mice (Figure S3B). After IAV infection, pipecolic acid treatment (75 mg/kg) attenuated weight loss (Figure 7B) and reduced lung virus titers to between 1/1.2 and 1/20 of the levels in PBS‐treated mice (*p* = 0.0468) (Figure 7C).

We next evaluated whether pipecolic acid confers broader protection against other IAV subtypes. Mice pretreated with pipecolic acid were infected with 10^4.5^ TCID_50_ of influenza A/Puerto Rico/8/1934 (H1N1) (referred to as H1N1 PR8). Pipecolic acid‐treated mice showed comparable weight loss to PBS‐treated mice from Day 1 to 5, but pipecolic acid‐treated mice recovered to their initial weights more rapidly than PBS‐treated mice (Figure 7D). Pipecolic acid treatments significantly reduced lung virus titers by approximately 33% compared to PBS treatment (*p* = 0.0233) at 3 dpi (Figure 7E). Upon infection of IAV H9N2 Ch01 or H1N1 PR8, clinical symptoms in the pipecolic acid‐treated mice, including piloerection, delayed responses, arched back, and lethargy, were alleviated compared to the PBS‐treated mice (Figure S3C,D).

Given that EcN pretreatment has been shown to regulate cytokine and chemokine expression (Figure 2F), we investigated whether pipecolic acid elicits similar effects. The results showed that pipecolic acid treatment significantly suppressed pro‐inflammatory cytokines (IL‐1β and IL‐8) in IAV‐infected mice at 3 dpi (Figure 7F), though this effect diminished by 5 dpi (Figure 7F). These findings suggest that pipecolic acid confers protection against IAV in mice, potentially by mitigating early‐stage hyperinflammation and preventing excessive tissue damage. To test the hypothesis, we performed hematoxylin and eosin staining on mouse lung tissues. At 7 dpi, PBS‐treated mice showed significant inflammatory damage, including extensive alveolar disruption, minor exudate accumulation in the cavity, and partial necrosis of lung tissues, and cavity formation (Figure 7G). In contrast, pipecolic acid‐treated mice displayed better preservation of alveolar structure integrity, with only mild inflammatory cell infiltration. These histological improvements strongly suggest that pipecolic acid can protect against IAV‐induced lung injury.

### Pipecolic acid inhibits IAV infection in A549

**Figure 8:**
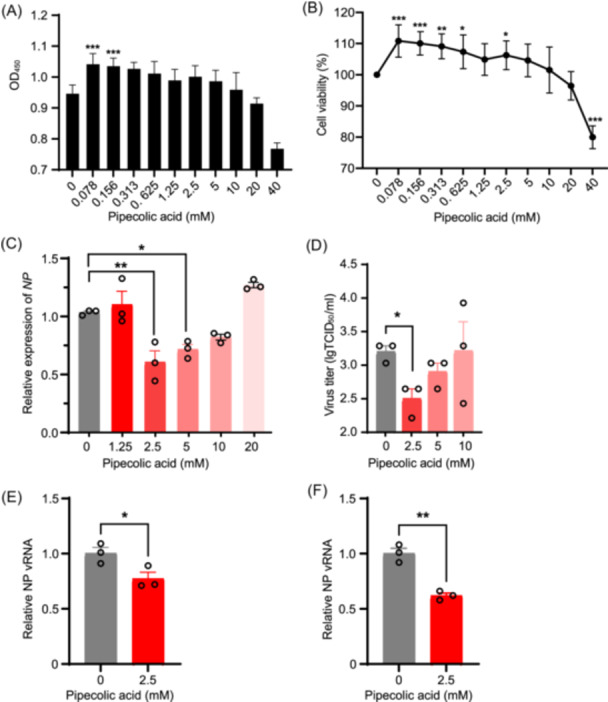
Cytotoxicity and anti‐influenza effects of pipecolic acid in vitro. (A, B) Assessment of cytotoxicity of pipecolic acid on the viability of A549 cells. A549 cells were incubated with varying concentrations of pipecolic acid for 48 h, and viability was determined by the CCK8 assay. (C) Pipecolic acid suppressing progeny virus production. A549 cells were infected with IAV H9N2 Ch01 at an MOI of 0.1 and incubated for 48 h with or without pipecolic acid. Cells were collected and *NP* expression was quantified by RT‐qPCR. (D) Culture supernatants collected to determine progeny virus titers using the TCID_50_ assay. (E) Examination of the viral entry by quantifying the level of NP viral RNA (vRNA) using qRT‐PCR. A549 cells were pretreated with pipecolic acid and then inoculated with IAV H9N2 Ch01. (F) Pipecolic acid blocks H9N2 viral entry in A549 cells. IAV H9N2 Ch01 were incubated with pipecolic acid and then inoculated into A549 cells to examine the viral entry by qRT‐PCR of NP vRNA. Data are presented as means ± SEM. *p < 0.05; **p < 0.01; and ***p < 0.001 (two‐tailed Student's *t*‐test).

To evaluate the biological effects of pipecolic acid, we first assessed its cytotoxicity in A549 cells using CCK8 assays. The calculated toxicity concentration 50 (TC_50_) value of pipecolic acid was 83.7 mM (Figure 8A,B). Subsequently, we selected 10 mM pipecolic acid as the maximum nontoxic dose to investigate its antiviral mechanism against IAV infection. We performed quantitative PCR (qPCR) and the TCID_50_ assay to assess the efficacy of pipecolic acid against IAV. The results demonstrated that pipecolic acid treatment significantly reduced IAV nucleoprotein (NP) expression (Figure 8C) and the virus titers (Figure 8D) in A549 cells induced with IAV H9N2 Ca01. To explore the mechanism by which pipecolic acid inhibits IAV infection in vitro, A549 cells were treated with pipecolic acid for 6 h before IAV inoculation. In parallel, IAV were pre‐incubated with pipecolic acid on ice for 2 h before inoculation into A549 cells. After incubation at 4°C for 3 h, the cells were washed three times with PBS and collected for viral RNA (vRNA) measurement. The results showed that pipecolic acid treatment significantly inhibited viral entry into the cells (Figure 8E,F). We also used an IAV mini‐genome assay to evaluate the effect of pipecolic acid on viral polymerase activity. Interestingly, pipecolic acid did not impair viral polymerase activity (Figure S4). The results demonstrate that pipecolic acid inhibits IAV infection in A549 cells, likely by blocking viral entry without affecting viral polymerase activity.

## DISCUSSION

EcN, the primary component of the probiotic formulation “Mutaflor^®^,” has been utilized as a pharmaceutical product to treat intestinal disorders for over a century, despite the general association of *E. coli* species with pathogenic potential^16^. EcN has also served as a platform for developing vaccines, biosensors, and various therapeutics, underscoring its well‐documented safety profile^36^. In this study, we demonstrated that oral administration of EcN conferred protection against influenza in mice, as evidenced by the significantly increased survival rate, reduced viral load, diminished inflammatory cell infiltration, and alleviated pathological injury. Analysis of 16S rDNA sequences from cecum contents revealed that EcN treatment significantly enhanced gut microbiota diversity, altered the F/B ratio, and enriched the growth of specific bacterial genera, such as *Bacteroides* and *Akkermansia*. Metabolomics profiling indicated that EcN facilitated the secretion of pipecolic acid, which contributed to defense against IAV infection. Furthermore, EcN appeared to regulate respiratory immune system by modulating cytokine expression. However, the antiviral effects of EcN were evaluated only against H1N1 and H9N2 IAV strains in this study. Given the genetic diversity and varying pathogenicity of IAVs, including differences in receptor‐binding preferences (e.g., α2,3‐ vs. α2,6‐linked sialic acids) and host adaptation mechanisms, these factors underscore the need for future studies to assess the efficacy of EcN against other clinically relevant IAV subtypes (e.g., H3N2, H5N1, or H7N9) and strains with pandemic potential^37^. Such investigations will clarify whether the observed protection is broadly applicable or strain‐specific.

Upon host cell invasion, IAV quickly replicates, suppressing host gene expression^38^ and causing host cell death via cytolytic or apoptotic mechanisms^39^. The death of infected cells triggers inflammatory responses^27^, upregulating expression of inflammatory, antiviral, and apoptotic genes, along with immune cell infiltration and tissue damage^40^. This cascade can lead to a “cytokine storm,” as observed in acute respiratory infections caused by highly pathogenic avian influenza viruses such as H5N1 and H7N9 IAVs, which cause significant immunopathology and severe disease^27^, ^41^. Huang et al. demonstrated that intranasal administration of EcN activates innate immunity in the respiratory tract to fight against influenza virus^25^. In our study, oral administration of EcN reduced lung IL‐8 levels and increased IL‐10 levels during IAV infection, suggesting that it protects against IAV infection and mitigate pulmonary pathological injury by curbing pro‐inflammatory cytokines. However, the immune effects of intranasal and oral administration may differ.

Wild mice possessing diverse gut microbiota show enhanced survival following H1N1 PR8 infection compared to laboratory counterparts, likely attributable to the evolved microbiota and immune system shaped by greater selective pressures from pathogens and inflammatory stimuli^42^. This highlights the established gut–lung axis linking gut microbiota and respiratory disease outcomes. In this study, EcN administration significantly increased the diversity of intestinal microorganisms in mice. *Firmicutes* and *Bacteroidota* were identified as the dominant phyla, collectively constituting approximately 90% of the intestinal microbiota^43^. The F/B ratio is widely recognized as a critical factor in maintaining intestinal homeostasis^34^. Compared to PBS‐treated mice, EcN pretreatment resulted in *Firmicutes* and *Bacteroidota* remaining the dominant microbial communities, comprising 90% of the gut microbiota. Furthermore, EcN administration increased *Firmicutes* abundance in the cecal contents, elevating the F/B ratio.

It has been reported that the abundance of the *Lachnospiraceae* NK4A136 group and *Lachnospiraceae* UCG 001 genera within the *Firmicutes* phylum notably increases, especially during the early phase of influenza virus infection (3 dpi). This early enrichment suggests their potential pivotal role in the host's resistance to IAV^44^, further supported by a negative correlation between the *Lachnospiraceae* NK4A136 group and intestinal permeability^45^. Within the *Bacteroidota* phylum, EcN pretreatment reduced the abundance of *Muribaculaceae*, *Alloprevotella*, and *Alistipes*. The fluctuations in *Muribaculaceae* abundance are mainly linked to diverse diets, host conditions, colonization, and gut dysbiosis^46^, ^47^. In a study comparing the saliva microbiome of Japanese oral cancer patients and healthy controls, *Alloprevotella* was found to be more abundant in individuals with oral cancer^48^. This observation aligns with previous research indicating that *Alloprevotella* enrichment may contribute to oral cancer development^49^. Within the *Verrucomicrobiota* phylum, EcN pretreatment increased the abundance of the *Akkermansia* genus. The role of *Akkermansia* in respiratory virus infections remains controversial, with studies reporting both protective effects against H7N9 IAV infection^50^ and detrimental effects during influenza virus infection^44^, necessitating further investigation to elucidate their specific contributions to host defense mechanisms.

The gut–lung axis mediates cross‐talk primarily via the systemic circulation of soluble microbial components and metabolites, immune cell migration, and the translocation of intestinal inflammatory mediators to the lungs^51^. Through metabolomic analysis, we identified several upregulated metabolites, among which pipecolic acid emerged as a non‐proteinaceous byproduct of lysine degradation. Pipecolic acid is a small molecule widely present in organisms, including plants, animals, and microorganisms, and it plays a crucial role in key physiological processes. In mammals, pipecolic acid shows tissue‐specific functions and participates in various physiological processes. For instance, in the mouse brain, pipecolic acid can cross the blood–brain barrier and potentially act as a γ‐aminobutyric acid (GABA) receptor agonist, which is essential for regulating brain activity and inhibitory neurotransmission^52^. Additionally, exogenous supplementation of pipecolic acid may offer potential benefits. For example, pipecolic acid supplementation attenuates pro‐inflammatory cytokine production in lipopolysaccharide (LPS)‐induced macrophages and relieves LPS‐induced sepsis in mice, indicating its anti‐inflammatory properties^53^. Early supplementation of l‐pipecolic acid significantly reduced depressive‐like behavior in mice with colitis and alleviated inflammatory cytokine levels in the colon, blood, and brain^54^. Furthermore, supplementation with l‐pipecolic acid alleviated faecal water content in C57BL/6 mice, enhanced intestinal transit, and improved constipation by increasing serum serotonin (5‐HT) and 5‐HT4 receptor (5‐HT4R) levels while reducing AQP3 levels^55^. These studies collectively highlight the multiple roles of pipecolic acid in physiological activities, which may explain why 2.5 mM pipecolic acid more effectively suppressed IAV replication compared to 5 mM pipecolic acid in cell culture. In this study, we also found that pipecolic acid protected mice from IAV H9N2 Ch01 and H1N1 PR8 IAV infections, alleviating clinical symptoms and pathological damage after infection. However, the origin of elevated pipecolic acid after EcN treatment remains unclear: is it derived from bacterial metabolism and host absorption, or induced by EcN administration? Further research is needed to track dynamic changes of pipecolic acid levels in peripheral blood plasma throughout influenza progression.

Pipecolic acid exists in two enantiomeric forms: l‐ and d‐pipecolic acid. l‐pipecolic acid, the natural l‐lysine metabolite, is synthesized via two distinct metabolic pathways: the Δ^1^‐piperideine‐2‐carboxylate (P2C) pathway or the α‐aminoadipic semialdehyde (α‐AASA)/Δ^1^‐piperideine‐6‐carboxylate (P6C) pathway^56^ . l‐pipecolic acid is primarily synthesized in microorganisms through the P2C, P6C, and lysine cyclodeaminase pathways. It functions as an osmoprotectant in *E. coli*, aiding bacteria growth under high‐osmolarity conditions.^57^ In mammals, l‐pipecolic acid is synthesized in the brain through the P2C pathway and accumulates in individuals with peroxisomal disorders, such as hyperpipecolatemia and Zellweger syndrome, where peroxisomes are deficient^58^. In addition, pipecolic acid is a metabolite of lysine, with the liver being the primary site of lysine catabolism in mammals^53^, ^59^ . d‐pipecolic acid concentration in human plasma significantly increases after the supplementation of soy germ extract, suggesting that the endogenous source of d‐pipecolic acid may primarily originate from the breakdown of dietary lysine by intestinal bacteria^60^. Therefore, it is speculated that the increased pipecolic acid levels are likely driven by host‐mediated synthesis through hepatic lysine catabolism, with EcN potentially amplifying these endogenous processes rather than relying solely on gut microbiota‐derived production. This enhancement correlates with alleviated influenza symptoms in mice. Nevertheless, the precise mechanisms by which pipecolic acid confers antiviral defenses require further elucidation.

## MATERIALS AND METHODS

### Mice

C57BL/6 and BALB/c mice (4 weeks old, female, specific pathogen‐free) were obtained from Slac Laboratory Animal Co. (Shanghai, China) and raised in the Experimental Animal Center of Zhejiang University. Mice were kept in environmentally controlled rooms (controlled room temperature: 25 ± 1°C) under 12 h light‐dark cycles, and provided sterile water and standard feed. All animal experiments were performed under Animal Biosafety Level 2 (ABSL2) conditions and were approved by the Institutional Animal Care and Use Committee (IACUC) of the Laboratory Animal Center, Zhejiang University (Approval No.: ZJU20220180).

### Cells and virus

Madin–Darby canine kidney (MDCK) cells were maintained in minimum essential medium (MEM, Gibco, NY) with 10% fetal bovine serum (FBS) (ExCell Bio, Shanghai, China). Human alveolar basal epithelial cells (A549) were cultured in F‐12 basic (Gibco, NY), and human embryonic kidney cells (293 T) were cultured in Dulbecco's modified Eagle medium (DMEM, Gibco, NY) with 10% FBS. All cells were cultured at 37°C under conditions of 5% CO_2_.

Influenza virus strains, A/Mink/China/01/2014 (H9N2) (referred to as H9N2 Ch01), A/California/04/2009 (referred to as H1N1 Ca04), and A/Puerto Rico/8/1934 (H1N1) (referred to as H1N1 PR8), were propagated in 10‐day. Specific pathogen free (SPF) embryonated chicken eggs were incubated at 37°C and 60% humidity for 72 h, and the allantoic fluid with viruses was stored at ‐80°C.

### Preparation and administration of Nissle 1917 or pipecolic acid in mice

EcN was cultured in Luria–Bertani (LB) medium at 37°C, supplemented with 1.5% agar when preparing agar plates. Probiotic acid concentration in a liquid suspension was measured at OD_600_ using a microplate spectrophotometer (Biotek Epoch). Mice received the designated amount of EcN (100 μl/mouse, 10^9^ CFU/ml) every other day via oral administration.

Pipecolic acid (CAS No.: 535‐75‐1, purity >98%, Aladdin) was diluted in PBS and administered to each mouse at a dosage of 75 mg/kg, with a volume of 100 µl per mouse. Oral gavage was conducted for 7 consecutive days, followed by intranasal infection with a specified viral dose.

### Viral infection of mice

Isopropanol‐anesthetized mice were intranasally infected with a median tissue culture infectious dose (TCID_50_) of influenza A virus (H9N2 Ch01, 10^4.5^ TCID_50_; H1N1 Ca04, 10^4.5^ TCID_50_; H1N1 PR8, 10^4.5^ TCID_50_) in 20 μl DMEM per mouse. Subsequently, changes in body weight and mortality were meticulously monitored for 14 consecutive days. As per ethical standards and regulations for animal experiments, the endpoint “mortality” was defined as weight loss exceeding 25% of the initial value^61^. At 0, 3, and 7 dpi, mice were killed, and relevant samples were collected for further analysis.

### Viral load determination

At specified time points, intact lungs and nasal washes were collected. The lungs were homogenized using a mechanical homogenizer (Servicebio, KZ‐III) in MEM supplemented with 2% BSA fraction V and 1% antibiotics. The viral supernatant that was harvested from lung tissue homogenates and nasal lavage following centrifugation, was subjected to various dilutions in MDCK cells (infected by TPCK‐trypsin) and detected in triplicate using the cytopathic effect (CPE) assay^62^. The calculation of TCID_50_ follows the method by Reed and Muench (1938)^63^.

### Clinical symptom score

Following IAV infection, clinical symptoms were observed and assessed using the following scoring criteria: 5. health (asymptomatic); 4. mild (slightly ruffled fur); 3. moderate (ruffled fur and hunching); 2. severe (ruffled fur, hunching, and shivering); and 1. Moribund (unresponsive to stimuli).

### Lung histopathology

At 7 dpi, following euthanasia of the mice, lung samples were dissected and fixed with 4% paraformaldehyde (Sinopharm Chemical Reagent Co., Ltd.) for 72 h. Subsequently, after fixation and embedding, 4‐μm‐thickness lung sample slices were obtained and stained with hematoxylin and eosin (RIBOLOGY Co., Ltd), dehydrated, and mounted for microscope observations. Photomicrographs (×400) for lung samples were captured using a NIKON DS‐U3 digital scanner.

### 16S rDNA sequencing of microbiome

The cecal contents of mice were collected promptly, snap‐frozen in liquid nitrogen, and stored at ‐80°C. Bacterial DNA was extracted from the fecal samples using Magnetic Soil and Stool DNA Kit (TianGen, Catalog #: DP712). The DNA concentration was determined via agarose gel electrophoresis and NanoDrop2000. Genomic DNA served as a template for PCR amplification of the V3–V4 region of the 16S rDNA using barcode‐specific primers 528F and 706R using the Phusion^®^ HighFidelity PCR Master Mix (New England Biolabs). Following PCR amplification, the products were purified by magnetic beads and subjected to electrophoresis on a 2% agarose gel for detection. Subsequently, the PCR products, mixed in equidensity ratios, were purified using the Universal DNA Purification Kit (TianGen, Catalog #: DP214). Equal amounts of the purified PCR products were pooled and subjected to sequencing. The library construction and sequencing were conducted by Novogene Biotech Co., Ltd.

### Metabolomics analysis

UHPLC‐LC‐MS/MS analyses were conducted utilizing a UHPLC system (Vanquish, Thermo Fisher Scientific) with a UPLC BEH Amide column (2.1 mm × 100 mm, 1.7 μm) coupled to a Q Exactive HFX mass spectrometer (Orbitrap MS, Thermo). The auto‐sampler temperature was 4°C, with an injection volume of 3 μl. A 50 μl sample was combined with 200 μl of extraction solution (acetonitrile:methanol = 1:1, containing an isotopically labeled internal standard mixture) in an EP tube. The mixture was vortexed, shaken for 30 s, sonicated for 10 min in an ice‐water bath, and incubated for 1 h at ‐40°C to precipitate proteins. Subsequently, the samples were centrifuged at 12,000 rpm, 4°C, for 15 min. The resulting supernatant was transferred to a fresh glass vial for analysis.

Data management involved several steps on the original data, primarily including noise removal through single peak filtration. Following data acquisition, principal component analysis (PCA) was conducted. The data were log‐transformed and mean‐centered using SIMCA software (V15.0.2, Sartorius Stedim Data Analytics AB), followed by automatic modeling analysis. Initially, the first principal component was subjected to OPLS‐DA modeling, and the model's quality was assessed through sevenfold cross‐validation. Then, model validity was evaluated by *R* ^2^ *Y* (the interpretability of the model to the classification variable *Y*) and *Q* ^2^ (the predictability of the model) after cross‐validation. Finally, a permutation test was used to obtain various random *Q* ^2^ values by altering the permutation order of the classification variable *Y* many times, further assessing the model. Differential metabolites were screened using Student's *t*‐test with a significance level of *p* < 0.05.

Regarding the correlation analysis of differential metabolites, the quantitative values of differential metabolites were used to calculate the Euclidean distance matrix for each group. Subsequently, differential metabolites were clustered using the complete linkage method and visualized through a heatmap. Comparative analysis of each group involved the categorization of different metabolites and the calculation of their association using the Spearman method. Finally, the chord graph was used for visual display.

In the functional analysis of differential metabolites, mapping of differential metabolites was performed using the KEGG (Kyoto Encyclopedia of Genes and Genomes, http://www.genome.jp/kegg/, and PubChem metabolite databases. By comprehensively analyzing the pathways housing differential metabolites (including enrichment analysis and topological analysis), key pathways with the highest correlation with metabolite differences were identified.

In the regulatory network analysis of differential metabolites, matching information of differential metabolites in each group was obtained, followed by a search in the KEGG database for related pathways and subsequent analysis of regulatory interaction networks.

### Metabolome and intestinal microbiota correlation analysis

The correlation between significant metabolites and bacterial groups was analyzed by the Spearman correlation coefficient using the MetOrigin tools^64^ available at https://metorigin.met-bioinformatics.cn/home/.

### Cytotoxicity assays

The cytotoxic effects of pipecolic acid on A549 cells were evaluated using CCK8 assays. Briefly, cells were seeded in 96‐well plates at 4 × 10^3^ cells/well with 100 μl of F12 medium and incubated overnight (37°C, 5% CO_2_). The treatment groups were exposed to either dimethyl sulfoxide (DMSO) or twofold serial dilutions of pipecolic acid (78 nM–40 mM) for 48 h. Subsequently, the cells were rinsed twice with PBS, stained with CCK8 for 1 h, and the plates were gently shaken. Absorbance at 450 nm was determined, and the TC_50_ values of pipecolic acid were calculated using the Reed–Muench method^63^.

### Antiviral activity assay

The antiviral activity of pipecolic acid against influenza viruses was assessed in A549 cells. A549 cell monolayers were cultured overnight in 6‐well plates. After rinsing twice with PBS, the cells were inoculated with A/Mink/China/01/2014 (H9N2) strains at an MOI of 0.1 for 2 h at 37°C. Subsequently, the viral inoculum was removed and replaced with diluted pipecolic acid in TPCK‐trypsin (2 μg/ml)‐supplemented medium, followed by a 48‐h incubation at 37°C. The viral titer in the supernatant was determined by the TCID_50_ assay. To examine the viral entry, A549 cells were treated with pipecolic acid for 6 h before IAV inoculation or IAV was incubated with pipecolic acid on ice for 2 h before inoculation into A549 cells. Then, the cells and virus were incubated at 4°C for 3 h, and the cells were washed with PBS three times and collected for vRNA measurement.

### RNA isolation and qRT‐PCR

A two‐step qRT‐PCR was used to examine specific mRNA levels. Following cell lysis, total RNA was isolated using the specified kit (Easy‐do Bio) as per the manufacturer's protocol. The concentration of RNA was measured using a spectrophotometer NanoDrop (Thermo Fisher), and 1 μg of total RNA was obtained for reverse transcription with HiScript II Q Select RT SuperMix for qPCR (+gDNA wiper) (Vazyme). Quantitative PCR was performed using the ChamQ Universal SYBR qPCR Master Mix (Vazyme) and run on a LightCycler® 480 II quantitative PCR system (Roche). RT‐PCR primers were designed using the PrimerQuest Tool. The sequences of primers for qRT‐PCR are detailed in Tables S2 and S3. Following triplicate amplification of each gene, the mean threshold (*C*_t_) values were calculated. *GAPDH* was used for normalization in gene expression analysis. Relative fold changes in gene expression across groups were determined using the $2^{-{\mathrm{\Delta \Delta C}}_{t}}$ method.

### Statistical analysis

All data were presented as mean ± SD from a minimum of three independent experiments. Statistical significance was assessed using a two‐tailed Student's *t*‐test and a two‐way ANOVA test. Differences between groups were considered significant if *p* < 0.05 (indicated with *), highly significant if *p* < 0.01 (indicated with **), and extremely significant if *p* < 0.001 (indicated with ***).

## AUTHOR CONTRIBUTIONS

**Di Wang**: Data curation; formal analysis; investigation; methodology; writing—original draft. **Longhai Yu**: Investigation; validation. **Qi Lu**: Investigation; methodology; validation. **Meiqing Han**: Investigation; methodology. **Baikui Wang**: Investigation. **Xianqi Peng**: Investigation. **Min Yue**: Resources. **Yan Li**: Conceptualization; funding acquisition; methodology; project administration; supervision; writing—review and editing.

## ETHICS STATEMENT

The protocols of the animal studies were approved by the Committee of the Laboratory Animal Center of Zhejiang University (ZJU20220180).

## CONFLICT OF INTERESTS

The authors declare no conflict of interests.

## Supporting information

Supporting information.

Supporting information.

Supporting information.

Supporting information.

Supporting information.

## Data Availability

Data for high‐throughput sequencing of the bacteria 16S rDNA have been deposited in the NCBI Short Read Archive (SRA) database under the BioProject identifier (ID) PRJNA1094629 (accession numbers SRR 30050198–30050217).
