# Supplementary figures and images for: Oral pretreatment with Escherichia coli Nissle 1917 enhances the host's defense against influenza A virus infection
Source: mLife. 2025 Dec 27;4(6):666–82. doi: 10.1002/mlf2.70050 (PMC12754630; doi:10.1002/mlf2.70050)

Supplementary Figure 1

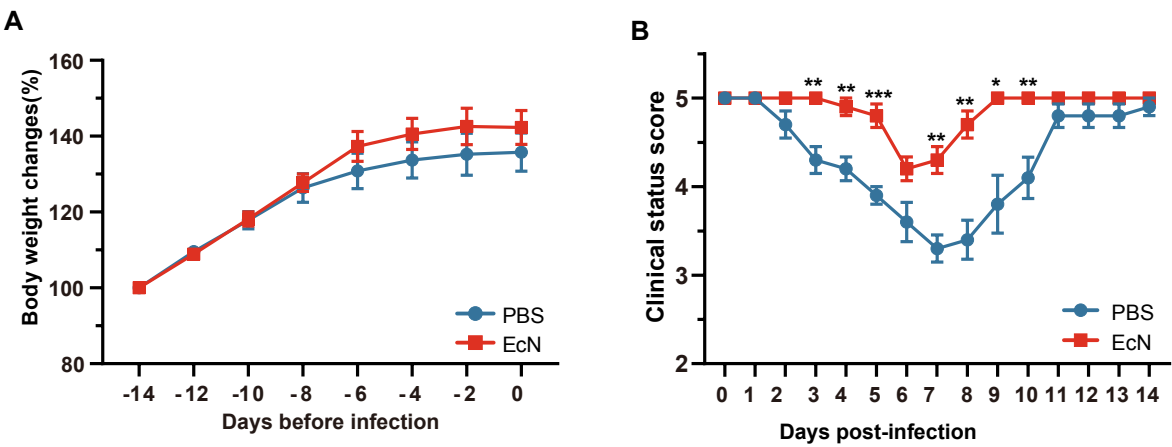

Supplementary Figure 2

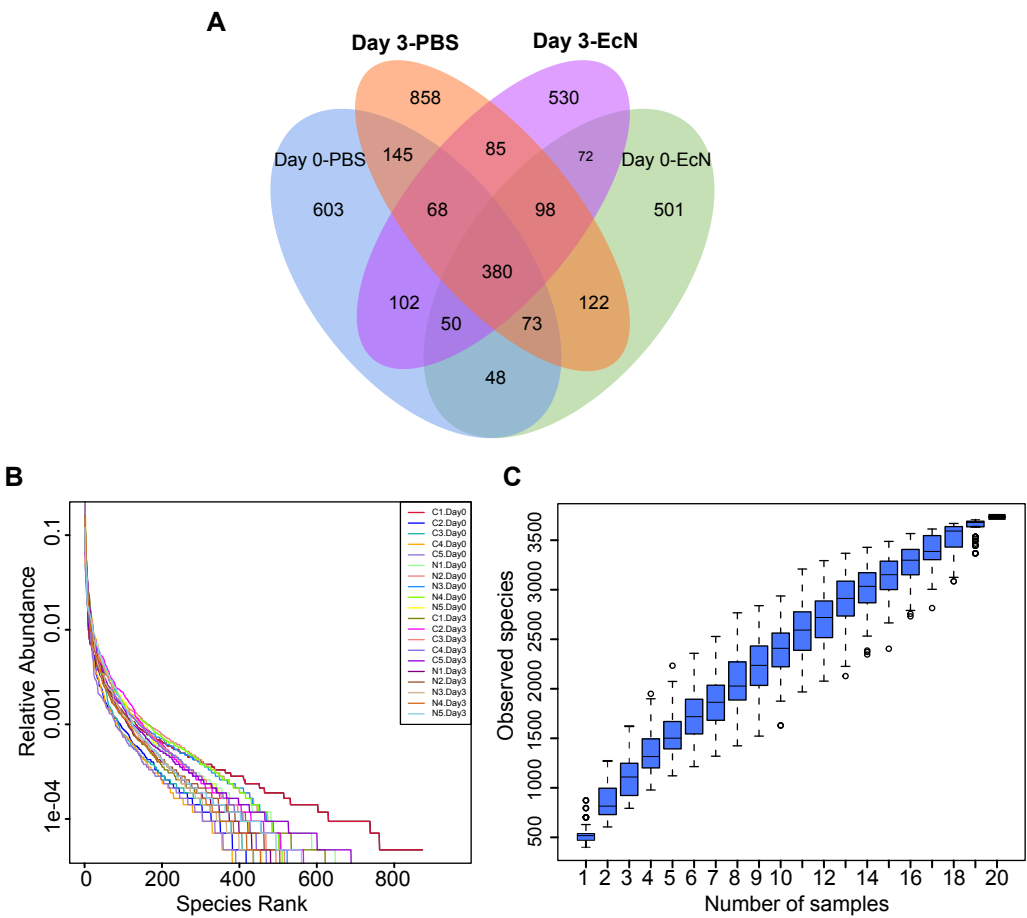

Supplementary Figure 3

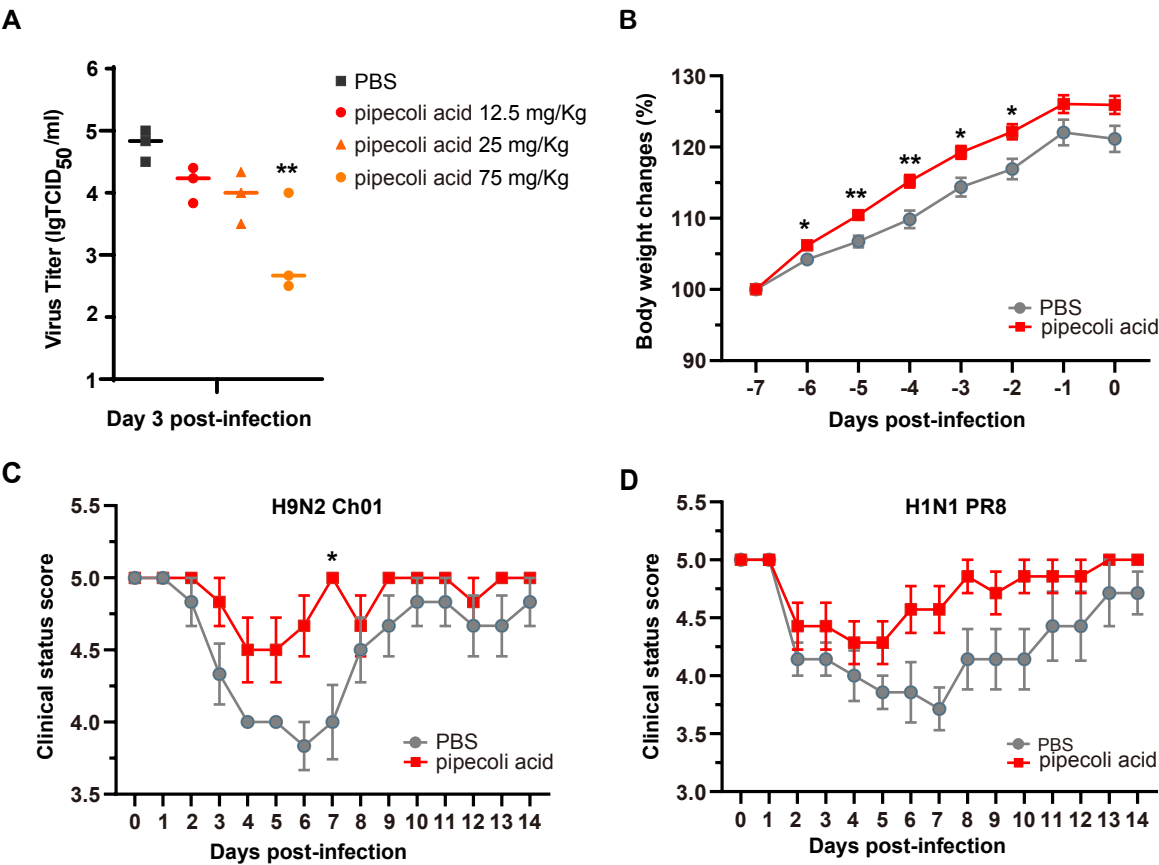

Supplementary Figure 4

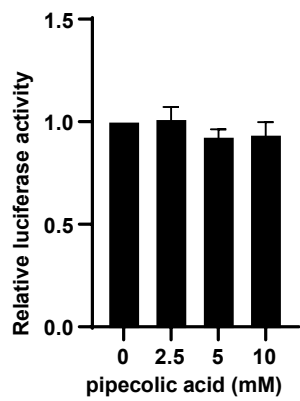

Supplement: Supplementary file 1 — Supporting information. [file MLF2-4-666-s003.pdf]
