# Supplementary material for: Oral pretreatment with Escherichia coli Nissle 1917 enhances the host's defense against influenza A virus infection
Source: mLife. 2025 Dec 27;4(6):666–82. doi: 10.1002/mlf2.70050 (PMC12754630; doi:10.1002/mlf2.70050)
Supplement: Supplementary file 5 — Supporting information. [file MLF2-4-666-s004.docx]

**Supplementary Figure 1. Mouse growth after oral administration of EcN, and relieved clinical symptoms caused by IAV infection in mice.**

**(A)** 4-week-old female BALB/c mice were oral administered EcN or PBS, and their body weights were measured daily post-administration. **(B)** Clinical symptoms were observed and scored following IAV H1N1 Ca04 infection based on the following scoring criteria: 5. Health (no clinical symptoms); 4. Mild (slightly ruffled fur); 3. Moderate (ruffled fur and hunching); 2. Severe (ruffled fur, hunching, and shivering); 1. Moribund (no reaction to the stimulation).

**Supplementary Figure 2. Modulation of EcN on the intestinal microbiology components by 16S rDNA sequencing.**

Fresh cecum samples were collected at 0 and 3 days post-infection and analyzed using 16S rDNA sequencing. The results included **(A)** the Wenn diagram, **(B)** rank-abundance, and **(C)** species accumulation boxplot.

**Supplementary Figure 3. Orally Administration of pipecolic acid enhanced mouse growth pre-IAV infection and alleviated influenza-induced clinical symptoms in mice.**

**(A)** Virus titers in the lung tissues collected on days 3 post IAV H9N2 Ch01 infection were measured by TCID_50_ assay (3 mice per group). **(B)** Body weight changes in BALB/c mice following pipecolic acid administration. Clinical manifestations were assessed and scored after infection with IAV H9N2 Ch01 **(C)** and H1N1 PR8 **(D)**.

**Supplementary Figure 4. Pipecolic acid did not enhance viral polymerase activity.**

**IAV mini-genome reporter assays were performed.** Data are presented as means ± SEM. *p < 0.05 (two-tailed Student’s *t*-test)

**Supplementary Table 1. Differential Metabolites.** The differential metabolites in the plasma of EcN-treated and PBS-treated mice.

**Supplementary Table 2. Mouse Primer Sequences Used in Quantitative Real-Time Polymerase Chain.**

**Supplementary Table 3. Human Primer Sequences Used in Quantitative Real-Time Polymerase Chain**
